# The role of iron metabolism as a mediator of macrophage inflammation and lipid handling in atherosclerosis

**DOI:** 10.3389/fphar.2014.00195

**Published:** 2014-08-27

**Authors:** Anwer Habib, Aloke V. Finn

**Affiliations:** Division of Cardiology, Department of Medicine, Emory University School of Medicine, Atlanta, GAUSA

**Keywords:** iron, macrophages, atherosclerosis, inflammation, lipid metabolism

## Abstract

Iron is an essential mineral needed for normal physiologic processes. While its function in oxygen transport and other important physiologic processes is well known, less is understood about its role in inflammatory diseases such as atherosclerosis. Existing paradigms suggest iron as a driver of atherosclerosis through its actions as a pro-oxidant capable of causing lipid oxidation and tissue damage. Recently we and others have identified hemoglobin (Hb) derived iron as an important factor in determining macrophage differentiation and function in areas of intraplaque hemorrhage within human atherosclerosis. Hb associated macrophages, M(Hb), are distinct from traditional macrophage foam cells because they do not contain large amounts of lipid or inflammatory cytokines, are characterized by high levels of expression of mannose receptor (CD206) and CD163 in addition to producing anti-inflammatory cytokines such as IL-10. Despite the well-known role of iron as an catalyst capable of producing lipid peroxidation through generation of reactive oxygen species (ROS) such as hydroxyl radical, we and others have shown that macrophages in areas of intraplaque hemorrhage demonstrate reduced intracellular iron and ROS which triggers production of anti-inflammatory cytokines as well as genes involved in cholesterol eﬄux. These data suggest that manipulation of macrophage iron itself may be a promising pharmacologic target for atherosclerosis prevention through its effects on macrophage inflammation and lipid metabolism. In this review we will summarize the current understanding of iron as it relates to plaque inflammation and discuss how further exploration of this subject may lead to new therapies for atherosclerosis.

## IRON IN THE VASCULATURE

Iron is a powerful catalyst resulting in the production of a hydroxyl radical through the oxidation of its ferrous (Fe^2+^) to ferric form (Fe^3+^) through the Fenton reaction (Crichton et al., 2002). In the endothelium, heme-derived iron is thought to catalyze oxidation of low density lipoproteins by itself or in conjunction with myeloperoxidase or lipoxygenase located on the endothelial surface (Balla et al., 1991; Miller et al., 1997; Camejo et al., 1998; Jeney et al., 2002; Yoshida and Kisugi, 2010). Hemolysis is often a result between the interaction of erythrocytes and mature atheromas resulting in the transition of ferrous to ferric forms of hemoglobin (Hb) which additionally leads to lipid oxidation (Nagy et al., 2010). Furthermore this oxidized form of Hb can also act as a pro-inflammatory agonist targeting vascular endothelial cells (Silva et al., 2009). Ferritin, a hepatic protein, may counteract some redox activity via ferroperioxidase in the vasculature (Balla et al., 1992), however, overall oxidation can be largely unchecked in these distinct pathologic environments as intraplaque hemorrhage. Oxidized low density lipoproteins (LDL) have a high affinity for the LDL receptor on macrophages leading their development into foam cells. Foam cells provide the major inflammatory component of atherosclerotic plaques. Foam cells density and necrotic core size within atherosclerotic plaques is thought to be a key determinant of plaque vulnerability for rupture (Sakakura et al., 2013). While the role of iron as a pro-oxidant has been established *in vitro* (Smith et al., 1992; Juckett et al., 1995; Pang et al., 1996; Silva et al., 2009), there is not a clear association of increased serum iron and increased incidence of coronary artery disease (CAD; Miller and Hutchins, 1994). In disease states of iron overload, such as hemochromatosis, an autopsy study found the extent of CAD to be less than the general population while another prospective study of 9000 individuals found carriers of the hemochromatosis genotypes C282Y to not have an increased risk for ischemic heart disease or myocardial infarction (Miller and Hutchins, 1994; Ellervik et al., 2005). Furthermore, dietary iron overload in Apo E^-/-^ mice reduces rather than exacerbates the severity of atherosclerosis (Kirk et al., 2001). These experimental data challenge the prevailing idea of iron as a pro-oxidant capable of accelerating coronary artery disease.

## SYSTEMIC IRON REGULATION AND LINKS TO INFLAMMATION

The majority of iron needed to regulate normal bodily functions is recycled from senescent red cells by the reticuloendothelial system. Additional demand for iron due to various environmental challenges such as anemia is fine-tuned by adjusting iron absorption via enterocytes. In some disease states, such as hemochromatosis, the regulation of iron is disturbed leading to excess iron entering the body. There are many systems within body that regulate the balance of iron. For the purposes of this review, we will focus on those within the macrophage. The regulation of movement of iron through various organs in the body is critical to maintaining iron homeostasis. Ferroportin (FPN), a transporter which mediates exit of iron from macrophages into the circulation, is an extremely important mechanism for immediate control of available and circulating serum iron. Although regulated at multiple levels, the peptide hormone, hepcidin, is the key regulator of FPN. Hepcidin binds to FPN inducing its internalization and degradation (Nemeth et al., 2004). Hepcidin induced downregulation of FPN thus inhibits cellular iron export from macrophages. The hepcidin-FPN axis is a major regulatory mechanism that maintains iron homeostasis in response to changing requirements. Also known as an acute phase reactant, hepcidin responds to inflammation resulting in adjustments to FPN levels which alters the regulation of body iron status (Ganz, 2003). The importance of this mechanism is observed in hereditary hemochromatosis where often either the expression or function of hepcidin is disturbed. In these situations, FPN is elevated because of low circulating hepcidin levels leading to increased gut iron absorption and pathologic deposition of iron in tissues.

Interestingly, mice deficient in the hemochromatosis gene, Hfe, have attenuated inflammatory responses to bacterial challenge associated with decreased macrophage TNF-α and IL-6 after exposure to the canonical Toll-like receptor 4 agonist lipopolysaccheride (LPS). These data suggest that these animals have impairment in Toll-like receptor 4 (TLR4) signaling (Wang et al., 2009). These defects could be replicated by exposing wild type murine macrophages to iron chelators, suggesting low intracellular iron within Hfe KO macrophage may lead to impaired TLR4 signaling. Thus, these results suggest iron overload in the setting of hemochromatosis may be associated with dampening of inflammation rather than exacerbating it.

## LOCAL IRON REGULATION BY MACROPHAGES AND LINKS TO ANTI-INFLAMMATION

In addition to helping to maintain systemic iron homeostasis, macrophages are intimately involved in preventing toxic effects of iron release during events involving hemolysis including in the setting of intraplaque hemorrhage. We and others have previously shown the importance of intraplaque hemorrhage, an event which leads to the deposition of erythrocyte-derived iron, in human atherosclerotic lesions (Kolodgie et al., 2003). In a relatively large number of human coronary plaques from sudden coronary death victims, we observed a greater frequency of previous intraplaque hemorrhages in plaques prone to rupture compared to early lesion morphologies or stable plaques. Hemorrhage itself contributes to the deposition of free cholesterol and enlargement of the necrotic core in atherosclerotic plaques through the accumulation of erythrocyte membranes that are rich in cholesterol. These findings were paralleled by an increase in macrophage density, which supports previous observations that hemorrhage itself is an inflammatory stimulus.

During hemorrhage, the pro-oxidant environment of atherosclerosis promotes erythrocyte lysis and accumulation of free Hb, which, if not eliminated, may cause tissue damage by releasing free iron which increases oxidative stress through the Fenton reaction. During hemolysis, free Hb binds to the plasma protein haptoglobin and hemoglobin:haptoglobin (HH) complexes are formed. CD163, the receptor for this complex, is expressed exclusively on the surface of macrophages and binds to HH, mediating its endocytosis. Conversely the interaction of haptoglobin itself with CD163 is impaired in highly oxidized environment (Vallelian et al., 2008), suggesting a more favorable interaction in the form of HH complexes. The heme subunit of Hb is then degraded by the heme oxygenase (HO-1) enzymes. The HO-1 pathway, which produces anti-oxidants carbon monoxide and biliverdin also releases free iron (Fe^2+^). Once iron has been released by HO-1, it is either utilized by the cell, stored as ferritin in a redox inactive form, or exported via FPN and converted to less redox active ferric iron (Fe^3+^) via ceruloplasm. Although the role of HO-1 in atherosclerosis has been studied in detail, an exact understanding of the molecular events in macrophages which orchestrate responses to iron and how this affects macrophage function remains incompletely understood. In addition, because hemorrhage, iron, and macrophages are not infrequently found in advanced atherosclerosis, the findings of these studies have important implications for our understanding of how iron itself event influences this disease.

The macrophage is the major inflammatory cell involved in atherosclerosis progression (Libby, 1995; Ross, 1999). While the role of lipid-rich foam cell macrophages which up-regulate proteolytic enzymes leading to plaque rupture has been extensively studied, less attention has been paid to alternative macrophage phenotypes which exist in atherosclerosis (Libby, 1995). It has been classically thought that macrophages exist in two subtypes: (1) “classically” activated (M1) macrophages, which are induced by Th1 cytokines such as tumor necrosis factor α (TNF-α) and LPS, and (2) alternative M2 cells, stimulated by Th2 cytokines such as IL-4 or IL-13 which produce anti-inflammatory cytokines such as IL-10 (Gordon, 2003).

Studies done by Boyle et al. (2009), in addition to our lab, suggest a third macrophage phenotype [M(Hb) or Mhem], induced by ingestion of HH complexes leading to an anti-inflammatory effect via production of anti-inflammatory cytokines such as IL-10 and production of anti-inflammatory metabolites produced during heme metabolism (Boyle et al., 2009; Finn et al., 2012).

## CD 163, INTRAPLAQUE HEMORRHAGE, AND MACROPHAGE POLARIZATION

Boyle et al. (2009, 2011) were the first to explore the effects of intraplaque hemorrhage on macrophage phenotype. Advanced atherosclerotic plaques were examined for immunostaining for CD163 and HLA-DR, a sign of macrophage activation. Macrophages were found to express either CD163 or HLA-DR. The CD163^high^ macrophages were found in areas of intraplaque hemorrhage and displayed evidence of less oxidative damage. This phenotype could be reproduced by exposure of human monocytes to HH complexes. More recently, our lab has expanded this work to demonstrate that macrophages in areas of human coronary intraplaque hemorrhage represent a subtype distinct from foam cells or the previously reported M2 phenotype.

**FIGURE 1:**
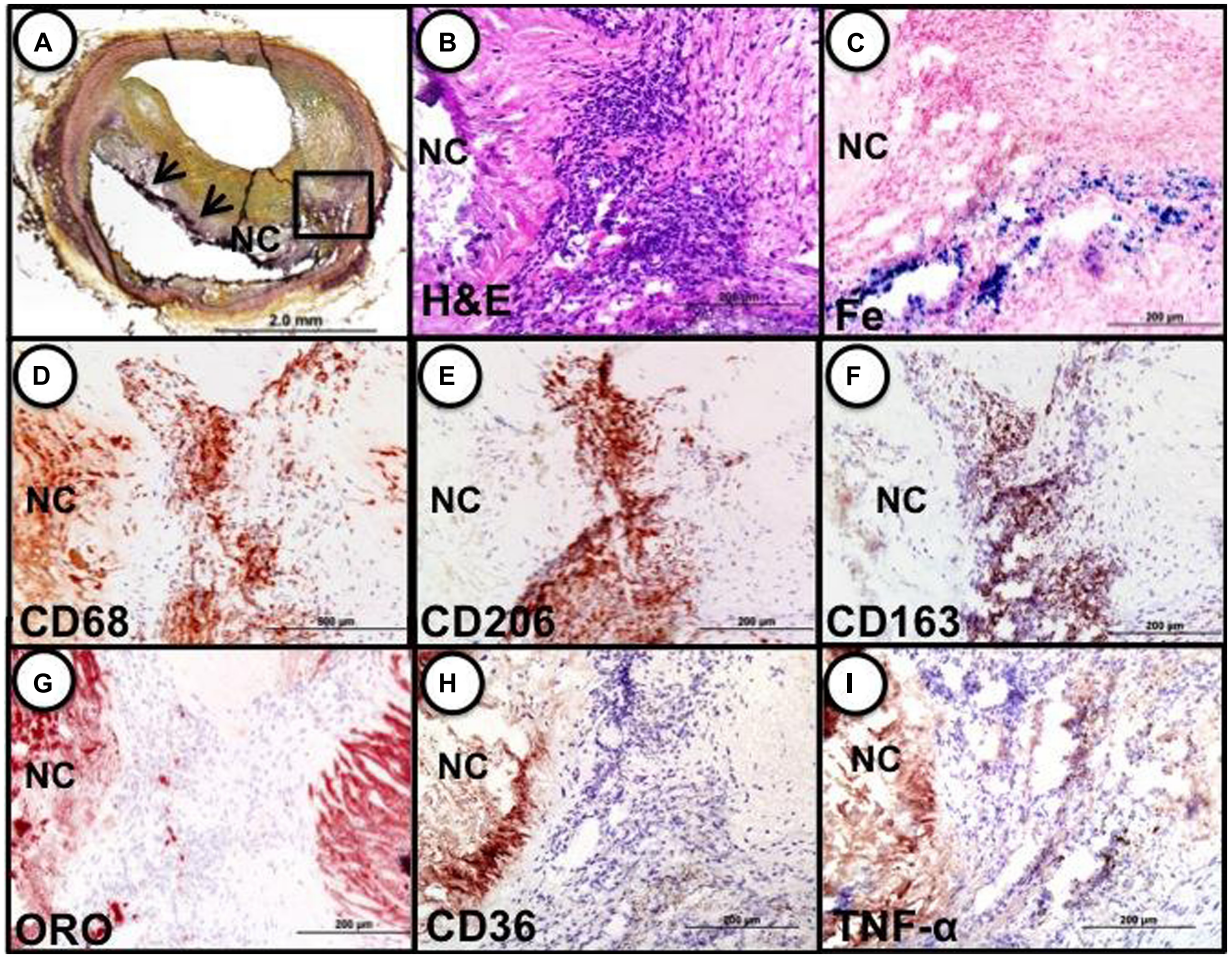
**Identification of M(Hb) macrophages in an area of hemorrhage in a human coronary fibroatheroma. (A)** Cryosection shows a fibroatheroma with a necrotic core (NC, arrows). Movat pentachrome staining. **(B–I)** represent the area within the black box in “a.” **(B)** Accumulation of inflammatory cells in an area of prior hemorrhage adjacent to the NC, H&E. **(E)** Iron (Fe) accumulation near the periphery of the necrotic core. **(D)** identification of macrophages by CD68 shows strong staining within the cell cluster adjacent to the necrotic core. **(E)** Intense staining for the mannose receptor (MR, CD206) within the cell cluster; note, however, the adjacent necrotic core shows negative staining. **(F)** The same MR positive macrophages within the cluster are also strongly positive for CD163, while the necrotic core remains negative. **(G)** Shows that the same cluster of cells is negative for lipid (ORO) while the adjacent necrotic core is strongly positive. The area of CD206/CD163 positive macrophages does not stain for CD36 **(H)** or TNFα **(I)**. Reproduced from Finn et al. (2012) permission pending.

**FIGURE 2:**
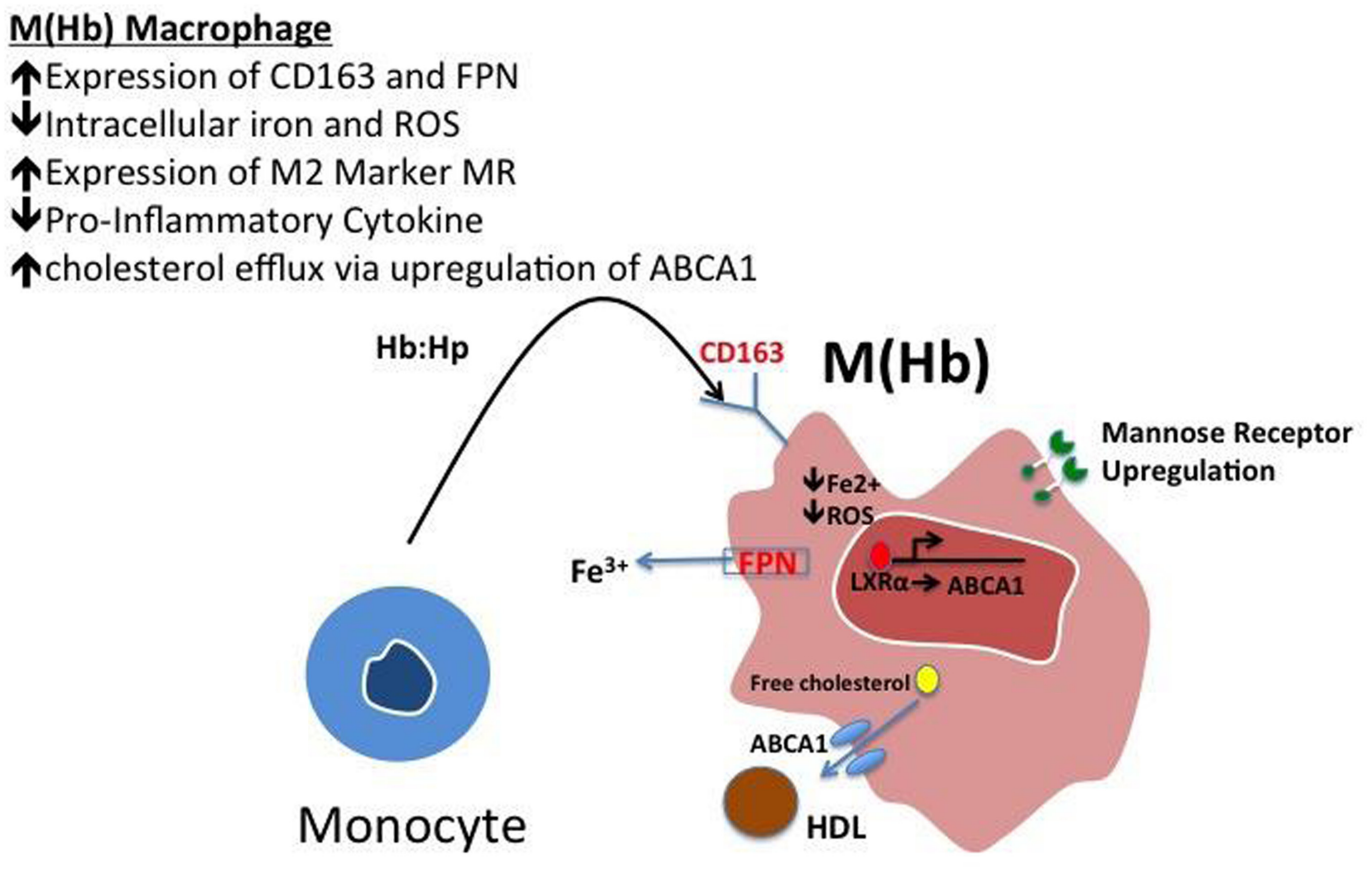
**Polarization of hemoglobin-associated macrophage, M(Hb).** Macrophage polarization to the M(Hb) phenotype via exposure to hemoglobin: haptoglobin (HH) complex involves the increased expression of CD163, the HH receptor, increased ferroportin-1 (FPN), an iron exporter resulting in decreased intracellular iron and reactive oxygen species (ROS). These cells are characterized by decreased inflammatory cytokine (i.e., TNF-α) expression in addition to increased reverse cholesterol transport via ABCA1, changes which are driven by reduced intracellular iron.

These cells, characterized by high surface mannose receptor (MR, CD206) and CD163, exhibit reduced expression of pro-inflammatory cytokines such as tumor necrosis factor alpha (TNFα), and are devoid of lipids typical of foamy macrophages (**Figure 1**; Finn et al., 2012). The term M(Hb) or Hb associated macrophages (Mhem) was used to refer to this subset since induced by ferrous Hb not IL-4 or hemorrhage (Bouhlel et al., 2007; Boyle et al., 2009). These cells demonstrate a unique iron handling signature associated with activation of the nuclear receptor liver × receptor alpha (LXRα), upregulation of ferroportin (FPN) and CD163. The activation of LXRα in addition to HO-1 was thought to be via oxidative stress from heme release and phosphorylation of activating transcription factor 1 (ATF-1; Boyle et al., 2012). Cultured human monocytes exposed to HH complexes have reduced free intracellular iron and reactive oxygen species (ROS) levels likely due to increased sequestration of iron by ferritin and by increased export of free iron outside the cell via FPN. This reduction in free iron and ROS could be reversed by pre-treating with cells with hepcidin, suggesting the importance of FPN in this effect. Moreover, M(Hb) macrophage demonstrate resistance to lipid loading, lowered expression of genes involved in lipid uptake (i.e., SR-A1, SR-A2, CD36, SR-B1) that characterize foam cells and increased reverse cholesterol through ATP binding cassette (ABC) transporters (i.e., ABCA1, ABCG1) involved in Apo-A1 cholesterol eﬄux to high density lipoproteins (HDL; **Figure 2**).

Our work suggests that iron itself does not result in increased oxidative stress and lipid retention with atherosclerotic plaque macrophages. Instead areas of hemorrhage demonstrate the opposite findings with little evidence of oxidative damage as assessed by 8- hydroxyguanine staining and diminished macrophage foam cell formation. To demonstrate the causal effect of lowering intracellular iron in the phenotype of M(Hb) cells, we treated HH differentiated macrophages with hepcidin and found that ABCA1 expression was significantly reduced. Moreover this was associated the downregulation of LXRα activity, a major transcriptional driver or ABCA1. This suggests the importance of macrophage intracellular iron levels driving cholesterol eﬄux in M(Hb) cells. Additionally differentiation of human macrophages with anti-oxidants such as superoxide dismutase (SOD) increased ABC transporter expression suggesting lowered ROS as a final common trigger for increasing cholesterol eﬄux. This suggests that manipulation of macrophage iron levels via the hepcidin-FPN axis represents a promising avenue to retard atherosclerosis development via up-regulation of macrophage cholesterol eﬄux.

## MACROPHAGE DIVERSITY IN HUMAN ATHEROSCLEROSIS – ROLE OF M(Hb) vs. M2 MACROPHAGES

Recent studies such as those from Chinetti-Gbaguidi et al. (2011) have looked IL-4 induced M2 macrophages in human atherosclerotic plaques. However, unlike M(Hb) where intraplaque hemorrhage provides a precipitant for its differentiation, the source for driving IL-4 remains unclear. Additionally, IL-4 differentiated M2 macrophages demonstrate mannose upregulation but not CD163 and do not demonstrate the same iron handling signature in that they show no increase in FPN expression and minimal changes in HO-1 and ferritin heavy chain (Bories et al., 2013). However, when M2 macrophages were exposed to iron, both FPN, HO-1, and LXRα-dependent genes such as ABCA1 were induced, mimicking the phenotype of M(Hb) macrophages. These data suggest, regardless of the stimulus (Hb or less physiologic FeCl_3_), iron is an essential factor driving the phenotype found in areas of intraplaque hemorrhage.

Hemoglobin: haptoglobin differentiated macrophages resist exogenous lipid loading to a much greater extent compared to IL-4 differentiated M2 macrophages and are characterized by an entirely different expression pattern of lipid handling genes (Boyle et al., 2012). However, M(Hb) demonstrated reduced expression of the scavenger receptors CD36 and increased expression of cholesterol eﬄux genes ABCA1/ABCG1, M2 macrophages demonstrate the opposite pattern with increased CD36 expression and reduced expression of ABCA1 and cholesterol eﬄux (Chinetti-Gbaguidi et al., 2011). Furthermore, a microarray analysis of 2400 genes showed a distinct gene transcriptome signature of M(Hb) versus M2 macrophages (Boyle et al., 2012).

Our work suggests that liver x receptor alpha (LXRα), an inducible transcription factor known to be important in human macrophage ABC transporter transcription, may play a central role in the response to heme-derived iron ingestion. LXRα can be activated by oxysterols which can also be produced by iron loading. The works of Bories et al. (2013) indicates LXRα appears to direct the upregulation of FPN and the repression of hepcidin, a protein which inhibits iron transport out of macrophages by degrading FPN. LXRα is likely a critical mediator of iron responses in macrophages especially M(Hb) with roles in lipid handling and inflammatory responses through transcriptional control of FPN/hepcidin.

## HEPCIDIN-FPN AXIS: MODULATION OF MACROPHAGE DIVERSITY TO IMPROVE ATHEROSCLEROTIC PROGRESSION

Given the link between macrophage the hepcidin→FPN axis, macrophage intracellular iron and the atheroprotective phenotype of M(Hb) we examined the effect of inhibitors of hepcidin on macrophage lipid metabolism (Yu et al., 2008; Saeed et al., 2012). Bone morphogenic protein-1 (BMP-1) signaling is involved in hepcidin gene transcription via SMAD 1/5/8 phosphorylation (Yu et al., 2008). BMP-1 inhibitors, such as dorsomorphin, and LDN, potently inhibit hepcidin production by blocking BMP-1 receptors, ALK 2/3/6 preventing its downstream effects on SMAD (Boergermann et al., 2010; Saeed et al., 2012). Effects of this BMP-1 inhibition on macrophage polarization lead to increased ABCA1/G1 expression, decreased cytokine and ROS production and increased FPN production (Saeed et al., 2012). These effects again were mitigated through hepcidin repletion (Saeed et al., 2012). Interestingly, LDN treatment delayed atherosclerotic progression in transgenic ApoE knockout mice and increased serum iron suggesting a potent effect in reducing intracellular iron content and plaque progression (Saeed et al., 2012). It must be stated, however, that inhibition of BMP signaling could reduce atherosclerosis via additional mechanisms not explored by us Derwall et al. (2012). However, the long-term effects of such manipulations which increase serum and likely tissue iron via up-regulation of FPN remains unclear. Given the pivotal role of hepcidin in regulating iron homeostasis, its chronic inhibition could potentially result in an iron overload-like state, which may limit the actual clinical adoption of such as strategy.

Further support for our data come from others work which has shown shown that overexpression of hepcidin both *in vitro* and *in vivo* murine ApoE carotid plaque model increases plaque instability especially in the setting of macrophage iron loading (Li et al., 2012). Additionally Wang et al. (2009) demonstrated that similarly targeted inhibitors of BMP signaling significantly attenuated infectious and non-infectious enterocolitis in a mouse model, again reinforcing the anti-inflammatory effect of this strategy which may be mediated in part through TLR4 inhibition (Wang et al., 2009). Given these findings, it suggests that the hepcidin-FPN axis is an important modulator of inflammation and determinant of macrophage polarization.

## CONCLUSION

Our knowledge of the effects of iron on inflammation and atherosclerosis continues to evolve. Recent studies on human atherosclerosis demonstrate that areas of intraplaque hemorrhage where iron is abundant demonstrate reduced ROS, tissue damage, lipid retention and inflammation. These data challenge existing paradigms that iron is a catalyst capable of producing ROS which accelerates atherosclerosis. Our data point to an important role for LXRα, FPN, hepcidin in controlling macrophage iron levels and thereby determining these cells lipid handing and inflammatory potential. These studies suggest that approaches to reduce intracellular macrophage iron that involve downregulation of hepcidin either directly (i.e., via shRNA) or indirectly (i.e., BMP-1 inhibitors) and may present a therapeutic benefit for advanced atherosclerotic lesions and perhaps other inflammatory conditions. However, given side effects that would occur by interfering with the FPN/hepcidin axis, more investigation is necessary to define this strategy of local modulation of inflammation to prevent atherosclerosis progression.

## Conflict of Interest Statement

The authors declare that the research was conducted in the absence of any commercial or financial relationships that could be construed as a potential conflict of interest.
